# Validity and reliability of a novel 3D ultrasound approach to assess static lengths and the lengthening behavior of the gastrocnemius medialis muscle and the Achilles tendon in vivo

**DOI:** 10.1007/s00167-022-07076-2

**Published:** 2022-07-29

**Authors:** Andreas Habersack, Thomas Zussner, Sigrid Thaller, Markus Tilp, Martin Svehlik, Annika Kruse

**Affiliations:** 1 Department of Orthopaedics and Trauma, Medical University of Graz, Auenbruggerplatz 5, 8036 Graz, Austria; 2 Institute of Human Movement Science, Sport and Health, University of Graz, Mozartgasse 14, 8010 Graz, Austria; 3 Institute of Psychology, University of Graz, Universitätsplatz 2, 8010 Graz, Austria

**Keywords:** Triceps surae, Accuracy, Reproducibility, Test–retest reliability, Ultrasonography

## Abstract

**Purpose:**

Human muscle–tendon units (MTUs) are highly plastic and undergo changes in response to specific diseases and disorders. To investigate the pathological changes and the effects of therapeutic treatments, the use of valid and reliable examination methods is of crucial importance. Therefore, in this study, a simple 3D ultrasound approach was developed and evaluated with regard to: (1) its validity in comparison to magnetic resonance imaging (MRI) for the assessment of the gastrocnemius medialis (GM) MTU, muscle belly, and Achilles tendon lengths; and (2) its reliability for static and dynamic length measurements.

**Methods:**

Sixteen participants were included in the study. To evaluate the validity and reliability of the novel 3D ultrasound approach, two ultrasound measurement sessions and one MRI assessment were performed. By combining 2D ultrasound and 3D motion capture, the tissue lengths were assessed at a fixed ankle joint position and compared to the MRI measurements using Bland–Altman plots. The intra-rater and inter-rater reliability for the static and dynamic length assessments was determined using the coefficient of variation, standard error of measurement (SEM), minimal detectable change (MDC_95_), and intraclass correlation coefficient (ICC).

**Results:**

The 3D ultrasound approach slightly underestimated the length when compared with MRI by 0.7%, 1.5%, and 1.1% for the GM muscle belly, Achilles tendon, and MTU, respectively. The approach showed excellent intra-rater as well as inter-rater reliability, with high ICC (≥ 0.94), small SEM (≤ 1.3 mm), and good MDC_95_ (≤ 3.6 mm) values, with even better reliability found for the static length measurements.

**Conclusion:**

The proposed 3D ultrasound approach was found to be valid and reliable for the assessment of the GM MTU, muscle belly, and Achilles tendon lengths, as well as the tissue lengthening behavior, confirming its potential as a useful tool for investigating the effects of training interventions or therapeutic treatments (e.g., surgery or conservative treatments such as stretching and orthotics).

**Level of evidence:**

Level II.

**Supplementary Information:**

The online version contains supplementary material available at 10.1007/s00167-022-07076-2.

## Introduction

Muscle–tendon units (MTUs) are elementary components of the musculoskeletal system [11]. MTUs and their components are highly plastic and undergo changes in response to, for instance, bone growth and mechanical loading, diseases, and disorders. In this context, information about individual muscle belly and tendon lengths is helpful to assess the presence of muscle belly contracture [3] and improves our understanding of structural adaptations [25]. Furthermore, gathering information about adaptations in muscle belly and tendon lengths due to treatments (e.g., stretching, orthotics) is important when developing efficient therapies. Consequently, the use of easily applicable, valid, and reliable methods to assess both the length and lengthening behavior of these tissues is of crucial importance.

For the assessment of muscle and tendon properties, ultrasound is often the method of choice since it has several advantages when compared with, for instance, magnetic resonance imaging (MRI) [20, 30–32]. However, assessing the length of a whole MTU is difficult with conventional 2D ultrasound. Therefore, various ultrasound approaches have been developed. For instance, assessment of the gastrocnemius medialis (GM) MTU, muscle belly, or (free) Achilles tendon has been performed using approaches combining: (1) B-mode ultrasound and 3D motion capture (e.g., [12, 14, 21, 36, 37]), and also 3D freehand ultrasound (3DfUS) approaches [2, 10, 28]; (2) ultrasound and tape measurements [3–5, 9, 15, 19, 25, 27]; and (3) extended field of view (EFOV) imaging/panoramic ultrasound [9, 13, 33, 35, 38, 40, 43, 44]. Although most of these approaches have been found to be accurate and reliable, their validity when compared with a gold standard, such as MRI has rarely been evaluated [2, 4, 35]. Furthermore, several drawbacks of these approaches exist, e.g., lying perfectly still is obligatory, 2D simplification of biological tissue properties.

In this study, to overcome the above-mentioned limitations, we developed an easily applicable 3D ultrasound approach that can be used to measure the lengths of the GM MTU, muscle belly, and Achilles tendon in 3D space by combining 2D ultrasound imaging, 3D motion capture, and vector algebra. The aim of this explorative study was to evaluate the validity of this approach. Furthermore, the intra-rater and inter-rater reliability was examined. We hypothesized that the proposed approach would have an adequate accuracy and high reliability for muscle–tendon length measurements.

## Materials and methods

The study was approved by the Ethics Committee of the University of Graz, Austria (registration number: 39/20/63 ex 2020/21). All participants (see the “Results” section) were informed about the study procedure, its purpose, and MRI safety. Written informed consent was obtained beforehand.

### Experimental design

To test the validity of the approach, ultrasound data, which were collected during an initial ultrasound measurement session, were compared with the MRI data captured 24.4 ± 8.1 h after the ultrasound assessment. Reliability analyses were conducted based on the ultrasound data captured during the initial and a second ultrasound measurement sessions (separated by 2.1 ± 0.6 days).

### Validity assessment

To test the validity of the 3D ultrasound approach, the static lengths of the GM MTU, the GM muscle belly, and the Achilles tendon were determined using both the proposed ultrasound procedure (see the “Reliability assessment” section) and MRI. Owing to contraindications (use of the contraceptive coil) and technical issues, six participants were excluded from the validity assessment.

The MRI data were acquired using a 3 T Magnetom Vida system with syngo MR XA20A software (Siemens Medical Systems, Erlangen, Germany) at the MRI-Lab at Graz University. The T1 measurements were obtained using a 3D space sequence derived from the commercial Numaris/X VA20A package. The full sequence protocol is provided in the Appendix.

Before scanning, two spherical markers (6-mm diameter, MR-PinPoint^®^, No. 187, Beekley Medical^®^) were placed with their center at the level of marks indicating the origin and insertion of the GM MTU determined during the first ultrasound measurement session, therefore, corresponding to the ultrasound scanning position. Two custom-made splints (Ortho-Aktiv; Graz, Austria) were then placed on the participant’s ankle joints to ensure the same joint position during all measurements. The right ankle joint was stabilized at 90° and the left ankle joint at 80° (i.e., 10° dorsiflexion).

The participant was positioned supine with the knees slightly flexed. The participant’s feet were placed in a Head/Neck-20 coil with two Body-18 coils covering the upper part of the measurement volume in an overlapping setup and a Spine-32 coil covering the lower part, ensuring a signal-to-noise ratio (SNR) optimized volume coverage from the soles of the feet to the middle of the thighs.

### Reliability assessment

To examine the intra- and inter-rater reliability, a randomization of the starting order for both investigators and the test legs was performed (www.randomizer.org) and a standardized protocol was used. The participant was instructed to lie prone on an examination bench. First, the static length measurements were performed, followed by the dynamic examinations.

#### Static length assessment

**Fig. 1:**
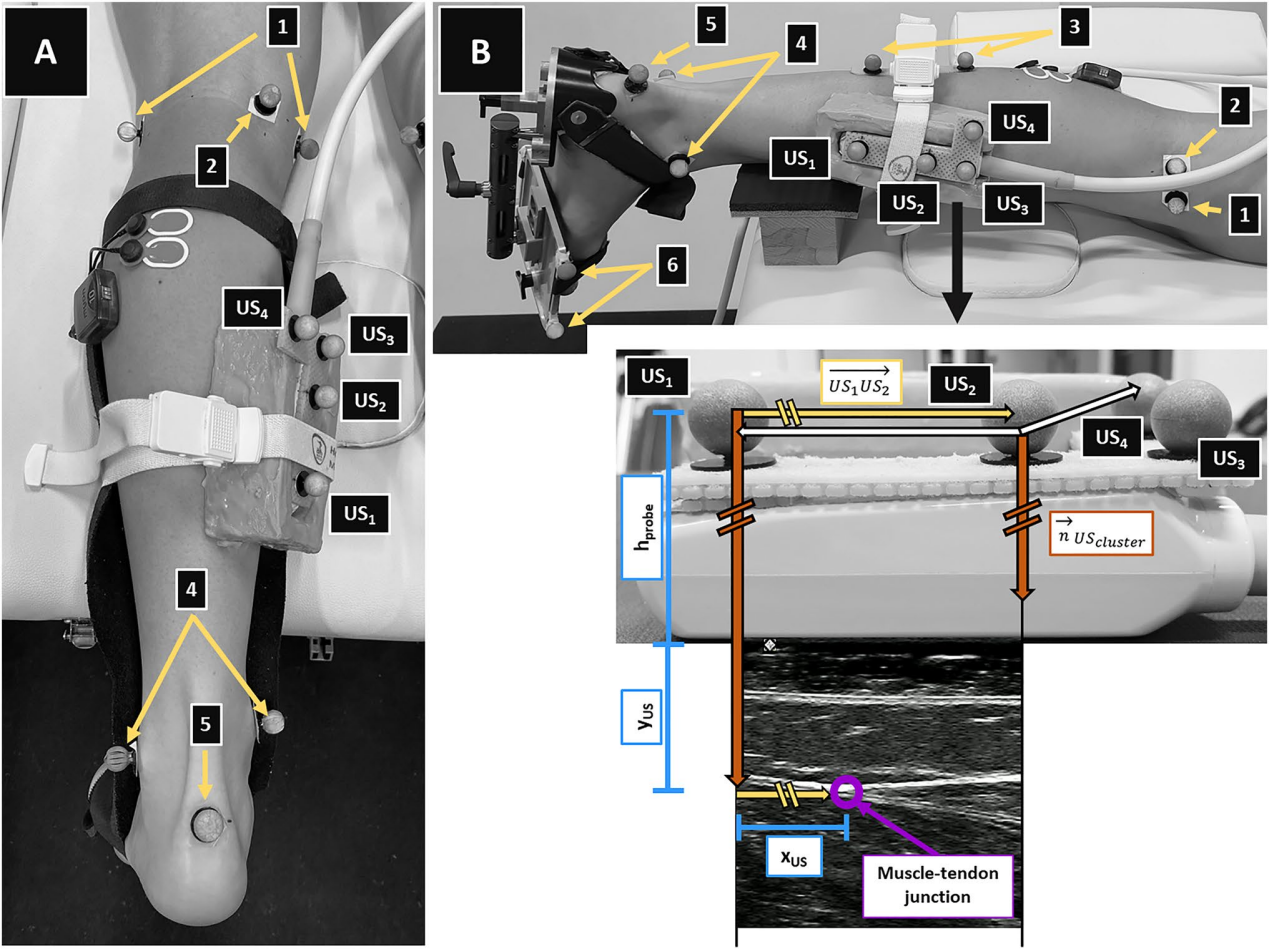
Measurement set-up for the static (**A**) and dynamic (**B**) trials. Placement of reflective markers, ultrasound transducer, and electromyographic sensors to assess gastrocnemius medialis muscle belly, tendon, and muscle–tendon unit lengthening behavior, and muscle activity throughout dorsiflexion rotations, respectively. Marker placement locations: 1 = medial and lateral condyle; 2 = most superficial point of the medial condyle; 3 = four-marker cluster; 4 = medial and lateral malleolus; 5 = proximal insertion of the Achilles tendon onto the calcaneus; 6 = four markers attached to footplate; US_1_ − US_4_ = markers placed on the ultrasound probe. **B** shows the procedure used to calculate the location of the muscle–tendon junction in vivo. Starting from marker US_1_, this point is corrected by the distance x_US_ along the horizontal direction, which is formed by vector US_1_US_2_, and then by the distance h_probe_ + y_US_ along the vertical direction, which is formed by the cross product of markers US_1_, US_2_, and US_4_

The ankle joint angle (Fig. 1A) was controlled with a goniometer (Ka We V01, Medizintechnik).

**Fig. 2:**
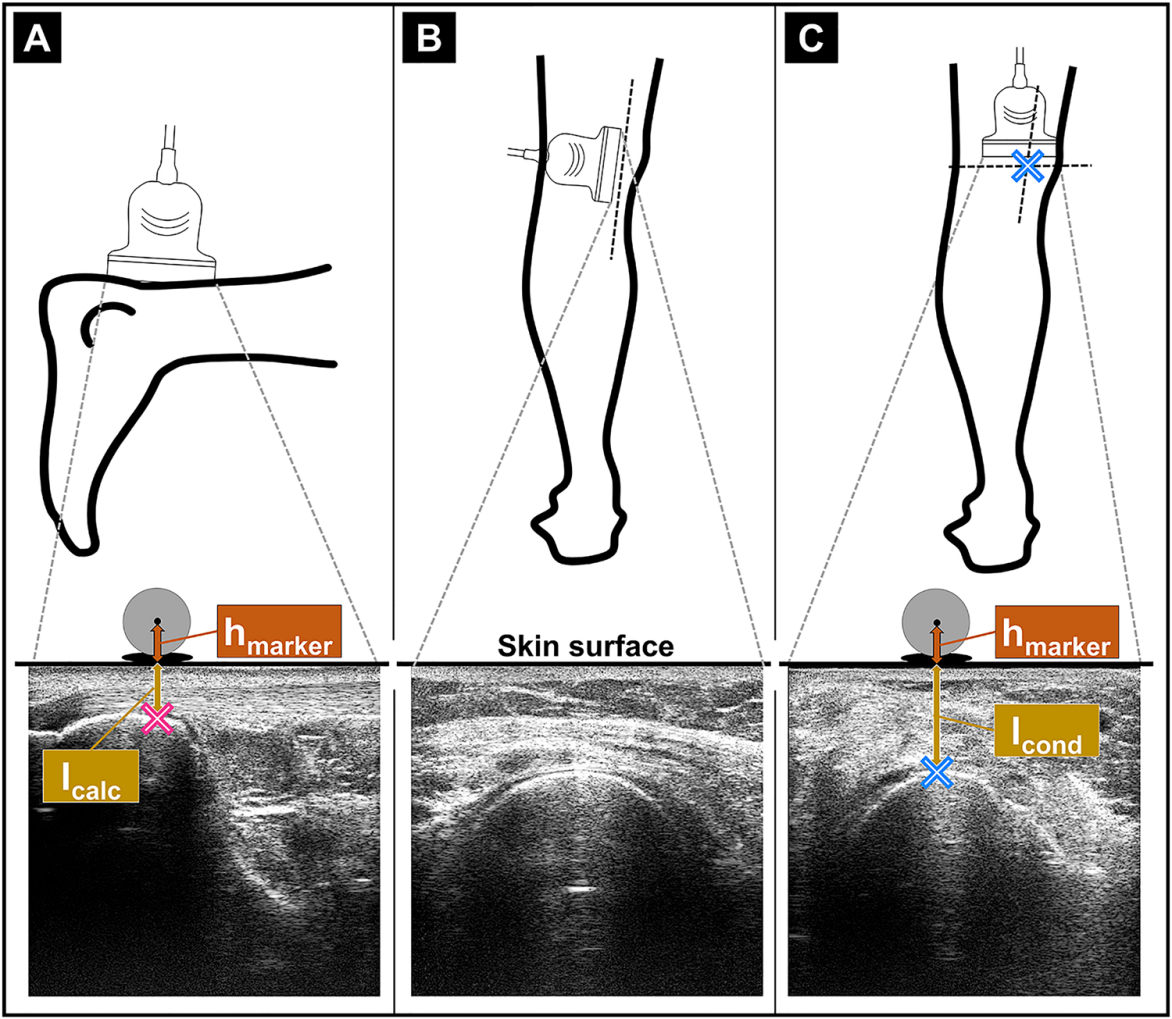
Determination of the anatomical landmarks. **A** The ultrasound transducer was placed in the longitudinal direction onto the heel to locate the proximal tendon attachment point at the calcaneus. The most superficial point of the femoral epicondyle was marked on the skin by localizing with the ultrasound probe in both the longitudinal (**B**) and transverse (**C**) planes. The reflective markers were placed collinear with the vertical axis above the determined landmarks. These markers were corrected along the vertical axis according to the distance from the center of the marker to the most superficial point of the medial condyle visible in the ultrasound images (h_marker_ + l_cond_). Accordingly, a correction was made according to the distance from the center of the marker to the proximal attachment point of the Achilles tendon at the calcaneus (h_marker_ + l_calc_)

First, a 5-cm linear array ultrasound transducer (LA523, MyLab 60; Esaote S.p.A., Genova, Italy) was used to detect the anatomical landmarks needed for the tissue length assessments (Fig. 2). The landmarks were the most superficial point of the medial epicondyle detected at the popliteal fossa, the GM muscle–tendon junction (MTJ), and the proximal tendinous insertion at the calcaneus (Fig. 2). The MTJ was determined by following the path of the muscle belly and visualizing its most distal point in the transverse plane. The anatomical sites were marked, and two ultrasound images were recorded showing the landmarks (Fig. 2). To avoid bias, the skin marks of the first investigator were removed before the second investigator started.

To control for possible muscle activation during the passive measurements, surface electromyographic (EMG) signals of the gastrocnemius lateralis were recorded (Fig. 1). Skin preparation and electrode placement (Blue Sensor N, Ambu A/S, Ballerup, Denmark) were carried out according to the SENIAM guidelines [17].

The reflective markers associated with the 3D motion capture system (10 cameras, Miqus M3, Qualisys AB, Gothenburg, Sweden) were placed on to predefined sites displayed in Fig. 1A, B. Moreover, a 59-mm linear array transducer (LogicScan 128; Telemed, Vilnius, Lithuania) fitted with a rigid cluster of four reflective ultrasound markers was fixed over the GM MTJ (Fig. 1A, B).

The marker positions and EMG data, as well as the ultrasound videos, were simultaneously recorded during two trials at 2000 Hz and 60 Hz, respectively. Afterwards, the second leg was prepared and measured as described above.

#### Dynamic lengthening assessment

After finishing the static measurements, the lengthening behavior of the GM MTU of the starting leg was assessed. The knee was placed at  ~ 20° flexion using a custom-made cushion (Fig. 1B) [6], and a custom-made footplate was applied to the foot (Fig. 1B) [18, 42]. Furthermore, an inclino-dynamometer [7, 18] was attached to the footplate, and the ultrasound transducer was placed onto the GM MTJ.

Altogether, two rotations were performed using the inclino-dynamometer to move the foot sole into dorsiflexion. The externally applied torque was simultaneously measured [42]. The displacement of the MTJ was recorded.

### Data analyses

#### Ultrasound examination

The MTJ displacement was manually tracked in the ultrasound videos [23]. The analysis of the tissue length and lengthening behavior was conducted using custom software programmed in MATLAB. To determine the absolute GM MTU, muscle belly, and tendon lengths, the origin and heel markers (Fig. 1) were corrected to determine the locations of the anatomical landmarks in vivo in 3D (Fig. 2A, C).

For the dynamic trials, the heel marker was corrected along the direction defined by the medially fixated footplate markers (Fig. 1B). Finally, the location of the MTJ was determined in 3D as displayed in Fig. 1B. The location of the MTJ was then determined using the calculated vectors, the ultrasound probe height (h_probe_), and the vertical (y_US_) and horizontal (x_US_) coordinates of the MTJ obtained from the tracking procedure.

Muscle belly length was computed as the linear distance between the corrected origin and the MTJ, and tendon length was calculated as the distance between the MTJ and the corrected heel marker position by applying the Pythagorean theorem. The MTU length was calculated as the sum of both lengths.

To assess the lengthening behavior, the individual GM muscle belly, tendon, and MTU lengths were calculated over the range from 0 Nm (i.e., resting length) to the maximum applied common torque (5.5 Nm) for all participants.

#### Magnetic resonance imaging examination

Two T1 space measurements were performed with overlapping FoVs and later combined into a single 3D volume, using the Numaris/X VA20A angio-compose algorithm. MRI evaluations of the combined 3D volumes were performed with Siemens View&GO in Siemens reference space.

The origin of the GM MTU was determined by locating the longitudinal and transverse slices with the largest marker diameter and 3D referencing the position in the transverse plane. The most superficial point of the medial epicondyle was determined and marked in the three spatial planes by scrolling through the referenced coronal slices. The proximal tendinous insertion of the GM MTU and the GM MTJ were also detected in the three spatial planes. Based on the marker positions, the muscle belly, tendon, and MTU lengths were directly measured in a single 3D volume for both legs (slice thickness of 1 mm).

### Statistical analyses

All the statistical analyses were performed using SPSS (version 22.0, SPSS Inc., Chicago, IL, USA). The level of significance was set to *α* = 0.05.

#### Sample size calculation

The sample size was based on the ICC estimates of Walter et al. [41]. To achieve an ICC of 0.9, a minimum acceptable ICC of 0.7, a power of 80%, and a significance level of 5%, the sample size calculation resulted in 18 subjects or legs. To account for any possible dropout, 16 healthy subjects (32 legs) were included, as in previous studies [3, 24, 33].

#### Validity

A dependent *t* test was used to compare the first ultrasound measurements (day 1) of both investigators, which were compared with the MRI analyses. Since the *t* test showed no significant difference (n.s.) between the raters, the collapsed ultrasound data (rater 1 + rater 2) were used. To assess the absolute agreement between the 3D ultrasound approach and MRI for the tissue lengths of both legs, Bland–Altman plots were utilized [8].

#### Reliability

For the reliability analyses, the coefficient of variation (CV) and intraclass correlation coefficient {ICC_(2,2)_ [95% confidence interval (CI)]} were used [21, 22, 24, 33, 34].

Moreover, the absolute reliability of the tissue lengths was evaluated by further calculating the standard error of measurement {SEM = SD × sqrt [1 − ICC_(2,2)_] [1, 16]}, where SD is the mean standard deviation of the respective test–retest length pair across the 15 participants [24]. The minimal detectable change (MDC) with a 95% CI was then derived [MDC_95_ = 1.96 × sqrt(2) × SEM [16]].

## Results

Sixteen healthy subjects were recruited for this study (Table 1), with 15 and 9 datasets finally used for the reliability (30 legs) and validity (18 legs) analyses, respectively. For the reliability assessments, the data of one participant were excluded due to the incorrect data recording. Participants refrained from any vigorous physical activity throughout the study participation.

**Table 1:** Participant characteristics (mean ± SD)

Sex	Number	Age (years)	Body mass (kg)	Body height (cm)	BMI
F/M	16	33.8 ± 8.4	73.0 ± 11.8	178.6 ± 8.8	22.9 ± 3.5
F	7	32.4 ± 10.5	67.0 ± 13.2	170.1 ± 4.6	23.1 ± 4.5
M	9	34.8 ± 7.2	77.7 ± 8.6	185.1 ± 4.4	22.7 ± 2.8

*SD* standard deviation; *F* female; *M* male; *BMI* body mass index

### Validity

The Bland–Altman plots (see Appendix) showed systematically smaller tissue lengths with the 3D ultrasound approach when compared with MRI, with differences of ≤  − 5.6 mm (max. error = 1.2%) and an average measurement error of 1.1% (for all tissues). The smallest difference was found for muscle belly length (≤ − 1.7 mm), followed by Achilles tendon length (≤ − 3.8 mm).

### Reliability

The mean CV of the static length assessments was ≤ 1.6% for all tissues (Table 2).

**Table 2:** Summary of the static ultrasound measurements, showing the mean (mm), standard deviation (SD, mm), coefficient of variation (CV, %) for sessions 1 and 2, and the intra- and inter-class correlation coefficients [ICC_(2,2)_] and their confidence intervals (95% CI), pooled standard deviation, standard error of measurement (SEM), and minimal detectable change (MDC_95_) for the left and right leg

	Session 1		Session 2		ICC_(2.2)_	95% CI	Pooled SD	SEM	MDC_95_
	Mean ± SD	CV	Mean ± SD	CV					
Left side									
Muscle belly length									
Intra-rater									
Rater 1	244.7 ± 24.5	0.12	242.2 ± 21.6	0.16	0.983	0.950–0.994	3.1	0.4	1.1
Rater 2	246.3 ± 22.2	0.12	242.3 ± 22.1	0.32	0.970	0.911–0.990	3.7	0.6	1.8
Inter-rater									
Pre	245.5 ± 23.2	1.20	–	–	0.987	0.963–0.995	2.9	0.3	0.9
Post	–	–	242.3 ± 21.7	1.32	0.984	0.953–0.995	3.1	0.4	1.1
Tendon length									
Intra-rater									
Rater 1	197.3 ± 31.1	0.17	194.3 ± 30.2	0.19	0.994	0.967–0.998	3.0	0.2	0.7
Rater 2	197.0 ± 29.0	0.13	197.6 ± 30.7	0.37	0.996	0.988–0.999	2.0	0.1	0.3
Inter-rater									
Pre	197.2 ± 30.0	1.18	–	–	0.995	0.987–0.998	2.3	0.2	0.5
Post	–	–	195.9 ± 30.4	1.58	0.993	0.956–0.998	3.0	0.3	0.7
MTU length									
Intra-rater									
Rater 1	442.1 ± 34.8	0.03	436.5 ± 34.8	0.03	0.994	0.978–0.998	2.9	0.2	0.6
Rater 2	443.2 ± 33.9	0.02	439.9 ± 33.8	0.03	0.994	0.983–0.998	2.9	0.2	0.6
Inter-rater									
Pre	442.7 ± 34.3	0.49	–	–	0.997	0.991–0.999	2.1	0.1	0.3
Post	–	–	438.2 ± 34.2	0.71	0.994	0.963–0.998	3.1	0.2	0.7
Right side									
Muscle belly length									
Intra-rater									
Rater 1	244.0 ± 23.8	0.13	240.3 ± 20.6	0.18	0.975	0.928–0.992	3.6	0.6	1.6
Rater 2	246.8 ± 21.3	0.21	243.4 ± 22.3	0.15	0.990	0.970–0.997	2.1	0.2	0.6
Inter-rater									
Pre	245.4 ± 22.4	1.55	–	–	0.981	0.941–0.994	3.7	0.5	1.4
Post	–	–	241.8 ± 21.3	1.32	0.982	0.929–0.995	3.2	0.4	1.2
Tendon length									
Intra-rater									
Rater 1	195.7 ± 32.8	0.20	193.3 ± 33.1	0.21	0.994	0.983–0.998	2.8	0.2	0.6
Rater 2	194.9 ± 33.9	0.25	194.8 ± 32.0	0.19	0.996	0.988–0.999	2.2	0.1	0.4
Inter-rater									
Pre	195.3 ± 33.3	1.12	–	–	0.996	0.990–0.999	2.2	0.1	0.4
Post	–	–	194.0 ± 32.5	1.07	0.997	0.990–0.999	1.9	0.1	0.3
MTU length									
Intra-rater									
Rater 1	439.7 ± 36.6	0.02	433.5 ± 35.8	0.02	0.993	0.973–0.998	3.5	0.3	0.8
Rater 2	441.6 ± 35.8	0.02	438.2 ± 33.7	0.02	0.996	0.988–0.999	2.3	0.1	0.4
Inter-rater									
Pre	440.7 ± 36.1	0.58	–	–	0.995	0.985–0.998	2.5	0.2	0.5
Post	–	–	435.9 ± 34.6	1.01	0.989	0.934–0.997	4.2	0.4	1.2

*Pre* results session 1; *Post* results session 2

The ICC values demonstrated excellent intra-rater and inter-rater reliability for all lengths and both sides, with ICC values of ≥ 0.97 (Table 2).

The SEM values ranged from 0.1 to 0.6 mm, and the MDC values were below 1.8 mm.

The mean CVs of the dynamic length assessments for both raters were ≤ 0.9% (Table 3). Slightly higher values were found for the inter-rater reliability, with the highest value for the muscle belly length (CV = 2.2%).

**Table 3:** Summary of the dynamic ultrasound measurements, showing the mean absolute tissue length changes (mm) calculated throughout a common torque interval (0–5.5 Nm), the coefficient of variation (CV in %) for sessions 1 and 2, the intra- and inter-class correlation coefficients [ICC_(2,2)_] and their confidence intervals (95% CI), and the pooled standard deviation (in mm), standard error of measurement (SEM in mm), and minimal detectable change (MDC_95_ in mm)

	Session 1		Session 2				ICC_(2,2)_		95% CI	Pooled SD	SEM	MDC_95_
	Mean ± SD	CV	Mean ± SD		CV							
Muscle belly												
Intra-rater												
Rater 1	16.9 ± 5.0	0.59	17.7 ± 5.2	0.52		0.983		0.946–0.994		2.7	0.4	1.0
Rater 2	15.5 ± 5.1	0.88	15.3 ± 6.2	0.85		0.977		0.928–0.993		3.2	0.5	1.4
Inter-rater												
Pre	16.2 ± 5.0	2.24	–	–		0.938		0.448–0.985		5.2	1.3	3.6
Post	–	–	16.6 ± 5.7	1.74		0.958		0.430–0.990		4.0	0.8	2.6
Tendon												
Intra-rater												
Rater 1	4.1 ± 2.8	0.35	3.5 ± 2.3	0.59		0.995		0.986–0.998		2.6	0.2	0.5
Rater 2	1.9 ± 1.9	0.58	1.2 ± 1.6	0.66		0.992		0.976–0.997		2.9	0.3	0.7
Inter-rater												
Pre	3.0 ± 2.6	1.82	–	–		0.988		0.964–0.996		3.7	0.4	1.1
Post	–	–	2.4 ± 2.3	1.38		0.994		0.981–0.998		2.8	0.2	0.6
MTU												
Intra-rater												
Rater 1	20.3 ± 5.5	0.24	21.2 ± 5.7	0.22		0.990		0.970–0.997		3.8	0.4	1.1
Rater 2	16.9 ± 4.4	0.29	16.5 ± 5.3	0.27		0.995		0.982–0.998		2.8	0.2	0.5
Inter-rater												
Pre	18.5 ± 5.2	1.40	–	–		0.974		0.770–0.994		6.0	1.0	2.7
Post	–	–	19.0 ± 5.9	1.26		0.982		0.376–0.997		5.4	0.7	2.0

*SD* standard deviation; *Pre* results session 1; *Post* results session 2

The ICC values demonstrated excellent intra-rater as well as inter-rater reliability for the length changes, with ICC values of ≥ 0.94 (Table 3). However, the inter-rater comparison led to a larger CI (Table 3).

The SEM values were small for both the intra-rater as well as inter-rater reliability, ranging from 0.2 to 1.3 mm.

The MDC values for the intra-rater reliability were below 1.4 mm for all the tissue length changes, while the inter-rater comparison led to higher MDC values of ≤ 3.6 mm.

## Discussion

The most important finding was that the 3D ultrasound approach showed good accuracy (mean error  ~ 1.1%) and excellent intra- as well as inter-rater reliability. When compared with MRI, the approach slightly underestimated the static lengths by 0.7%, 1.5%, and 1.1% for the muscle belly, tendon, and MTU lengths, respectively. Excellent intra-rater and inter-rater reliability was demonstrated by the high ICC (≥ 0.94), low SEM (≤ 1.3 mm), and good MDC_95_ (≤ 3.6 mm) values. Even better reliability was found for the static tissue lengths (e.g., SEM ≤ 0.6 mm; MDC_95_ ≤ 1.8 mm).

### Validity

To the best of our knowledge, this study is the first to have presented a 3D ultrasound approach and its validation for length measurements of the whole GM MTU, including separate assessments of muscle belly and tendon lengths. Although several studies have investigated the reliability of 2D ultrasound approaches, only a few have determined their validity by comparison with a gold standard [2, 4, 35]. For instance, Barber et al. [2] demonstrated the high validity of their 3DfUS approach, which slightly underestimated the GM muscle belly length by 3.3 mm (1.1%). Furthermore, Barfod et al. [4] reported good correlation between their ultrasound measurement and MRI underestimating tendon length by 4 mm. Silbernagel et al. [35, 36] demonstrated the high validity of EFOV imaging (ICC = 0.895) and a hybrid motion capture/ultrasound method (error < 1%) when compared with cadavers or a lamb shank, respectively, with shorter tendon lengths determined with the respective ultrasound method. Similarly, the proposed 3D ultrasound approach underestimated the muscle belly and tendon lengths by  ~ 1.6 mm (0.7%) and  ~ 3.3 mm (1.5%), respectively. The observed differences in tissue lengths between ultrasound and MRI may result from the difficulties in imaging the crucial anatomical sites. Although clearly identifiable in the MRI images in the three planes, ultrasound probe positioning and pressure may directly affect the determination in ultrasound images.

### Reliability

#### Static length assessment

Various reliable ultrasound approaches exist for measuring GM muscle belly and tendon lengths under static conditions. For instance, Silbernagel et al. [36] combined B-mode ultrasound and motion capture to assess Achilles tendon length, and reported excellent test–retest reliability, with ICC, SEM, and MDC_95%_ values of 0.97, 4 mm, and 11 mm, respectively. Similar values were found for tendon and muscle belly length by Cenni et al. [10, 11], who compared an ultrasound pointer method and their 3DfUS method.

When compared with these studies, the proposed 3D ultrasound approach resulted in slightly better reliability. In summary, we found ICC, CV, SEM, and MDC values of ≥ 0.97, ≤ 1.6%, ≤ 0.6 mm, and ≤ 1.8 mm, respectively, for the intra-rater and inter-rater reliability of the GM muscle belly and Achilles tendon lengths. The reliability of the GM MTU length measurements was even higher. Similar reliability results have only been reported for the (free) Achilles tendon using the Copenhagen Achilles length measure [15] or 3DfUS [28]. The MDC values found in the present study suggest that differences of  ~ 2 mm are needed for length changes to be considered real changes [16]. This finding is important for investigations of therapeutical treatment and/or training effects. The proposed 3D ultrasound approach could also be used to detect muscle and/or tendon alterations occurring due to or pointing at specific disorders/diseases (e.g., cerebral palsy).

Other approaches [3, 4, 9, 19, 40] have also shown excellent reliability. Using the approach of Barfod et al. [4], Brouwer et al. [9] investigated its reliability in comparison to Achilles tendon length measurements performed with EFOV assessments reporting better reliability for the ultrasound/tape approach [9]. In addition, Ryan et al. [33] examined the test–retest reliability of Achilles tendon length assessments performed with panoramic ultrasound. Although an excellent ICC value of 0.95 was reported, the SEM and MDC values were quite high (4.4 mm and 12.3 mm, respectively). In contrast, high test–retest reliability (SEM: 0.7 mm; ICC: 0.94; MDC_95%_: 1.8 mm) for tendon measurements performed with EFOV imaging were also reported [35]. Although Ryan et al. [33] concluded that panoramic ultrasound assessment can reliably detect changes in length, as seen in many clinical scenarios, their results may also indicate a need for experience with EFOV/panoramic ultrasound. Therefore, the use of a length assessment approach, as presented in this study, might be preferable.

#### Dynamic lengthening assessment

Ultrasound approaches appear to be a reliable way to assess muscle–tendon tissue lengths at static positions. However, they might be unsuitable for evaluating dynamic behavior. Because length adaptations may also impact mechanical tissue behavior, assessment of length changes is also crucial.

Although combined approaches of 2D ultrasound and 3D motion capture are often used for the measurement of muscle and tendon length changes in training [21] and clinical studies [39], their reliability has rarely been evaluated. Nakamura et al. [29] examined the test–retest reliability of GM MTJ displacement and reported high reliability (ICC = 0.99, pre–post difference = 0.8%) with the location of the MTJ only determined in 2D. Others [e.g., 21, 26, 39] did not report such reliability measures. In this study, we found high intra-rater reliability for the assessment of length changes of the GM MTU. Therefore, we assume that the proposed approach could reliably be used by one rater in clinical or training studies. Moreover, to the best of our knowledge, the proposed 3D approach is the first to calculate the position of the GM MTJ in 3D, independent of the ankle joint position or any prerequisites for probe placement, which could be beneficial.

When concerning inter-rater reliability, we also found excellent reliability, with CV, SEM, and MDC values of ≤ 1.8%, ≤ 1.0 mm, and ≤ 2.7 mm, for tendon and MTU length changes. The reliability for muscle belly length changes was only slightly lower. However, a larger range in CI values was detected for muscle belly and MTU changes. We assume that the differences between raters in the handling of the measurement apparatus caused by differences in anthropometrics (e.g., body height, hand size) might have influenced the outcomes. Further investigations are therefore needed.

The present study has some limitations. The subject positioning (MRI: supine; ultrasound: prone), which may affect the passive forces acting on the muscle belly despite the same joint configuration [2], might have affected the results. However, owing to the small differences observed in comparison to MRI, this factor might be negligible. Furthermore, evaluation was only performed for the GM MTU, and validations for other muscles/muscle groups should be performed in future studies. Finally, although a necessary sample size was reached (i.e., 18 legs for validation and 30 legs for reliability were measured) and valid and reliable results were determined, the results should be considered with caution. A greater sample size is needed to support the findings, therefore, the explorative character of the present study is stressed. However, based on the results, the potential of the proposed 3D ultrasound approach is also emphasized.

## Conclusions

The proposed 3D ultrasound approach was found to be valid and reliable for the assessment of muscle–tendon lengths, and the tissue lengthening behavior. Although the validation was based on a small sample size, the results of this explorative study support the fact that the presented approach could be used for the assessment of, for instance, the effects of short- and long-term training interventions or therapeutical treatments on the GM MTU in healthy as well as clinical populations.

## Supplementary Information

Below is the link to the electronic supplementary material.

Supplementary file 1 (PDF 79 KB)

Supplementary file 2 (PNG 564 KB)

Supplementary file 3 (DOCX 13 KB)

## Abbreviations

CI: Confidence interval
CS: Compressed sensing
CV: Coefficient of variation
EFOV: Extended field of view imaging
EMG: Electromyographic/electromyography
FoV: Field of view
GM: Gastrocnemius medialis
ICC: Intraclass correlation coefficient
MDC: Minimal detectable change
MRI: Magnetic resonance imaging
MTJ: Muscle–tendon junction
MTU: Muscle–tendon unit
SEM: Standard error of measurement
SNR: Signal-to-noise ratio
SPSS: Statistical Package for Social Sciences
3DfUS: Three-dimensional freehand ultrasound
2D: Two-dimensional
3D: Three-dimensional

## References

[1] Atkinson G, Nevill AM (1998). Statistical methods for assessing measurement error (reliability) in variables relevant to sports medicine. Sports Med.

[2] Barber L, Barrett R, Lichtwark G (2009). Validation of a freehand 3D ultrasound system for morphological measures of the medial gastrocnemius muscle. J Biomech.

[3] Barber L, Barrett R, Lichtwark G (2011). Validity and reliability of a simple ultrasound approach to measure medial gastrocnemius muscle length. J Anat.

[4] Barfod KW, Riecke AF, Boesen A (2015). Validation of a novel ultrasound measurement of Achilles tendon length. Knee Surg Sports Traumatol Arthrosc.

[5] Barfod KW, Riecke AF, Boesen A (2018). Validity and reliability of an ultrasound measurement of the free length of the Achilles tendon. Dan Med J.

[6] Bar-On L, Kalkman BM, Cenni F (2018). The relationship between medial gastrocnemius lengthening properties and stretch reflexes in cerebral palsy. Front Pediatr.

[7] Bénard MR, Jaspers RT, Huijing PA (2010). Reproducibility of hand-held ankle dynamometry to measure altered ankle moment-angle characteristics in children with spastic cerebral palsy. Clin Biomech.

[8] Bland JM, Altman DG (1986). Statistical methods for assessing agreement between two methods of clinical measurement. Lancet.

[9] Brouwer EF, Myhrvold SB, Benth JŠ (2018). Ultrasound measurements of Achilles tendon length using skin markings are more reliable than extended-field-of-view imaging. Knee Surg Sports Traumatol Arthrosc.

[10] Cenni F, Monari D, Desloovere K (2016). The reliability and validity of a clinical 3D freehand ultrasound system. Comput Methods Programs Biomed.

[11] Cenni F, Schless S-H, Bar-On L (2018). Can in vivo medial gastrocnemius muscle-tendon unit lengths be reliably estimated by two ultrasonography methods? A within-session analysis. Ultrasound Med Biol.

[12] Cenni F, Bar-On L, Schless S-H (2018). Medial gastrocnemius muscle-tendon junction and fascicle lengthening across the range of motion analyzed in 2-D and 3-D ultrasound images. Ultrasound Med Biol.

[13] Franchi MV, Fitze DP, Hanimann J (2020). Panoramic ultrasound vs. MRI for the assessment of hamstrings cross-sectional area and volume in a large athletic cohort. Sci Rep.

[14] Fry NR, Childs CR, Eve LC (2003). Accurate measurement of muscle belly length in the motion analysis laboratory: potential for the assessment of contracture. Gait Posture.

[15] Hansen MS, Kristensen MT, Budolfsen T (2020). Reliability of the Copenhagen Achilles length measure (CALM) on patients with an Achilles tendon rupture. Knee Surg Sports Traumatol Arthrosc.

[16] Hars M, Herrmann FR, Trombetti A (2013). Reliability and minimal detectable change of gait variables in community-dwelling and hospitalized older fallers. Gait Posture.

[17] Hermens HJ, Freriks B, Disselhorst-Klug C (2000). Development of recommendations for SEMG sensors and sensor placement procedures. J Electromyogr Kinesiol.

[18] Huijing PA, Benard MR, Harlaar J (2013). Movement within foot and ankle joint in children with spastic cerebral palsy. A 3-dimensional ultrasound analysis of medial gastrocnemius length with correction for effects of foot deformation. BMC Musculoskelet Disord.

[19] Intziegianni K, Cassel M, König N (2015). Ultrasonography for the assessment of the structural properties of the Achilles tendon in asymptomatic individuals: an intra-rater reproducibility study. IES.

[20] Jacobson JA (2005). Musculoskeletal ultrasound and MRI: which do I choose?. Semin Musculoskelet Radiol.

[21] Kay AD, Blazevich AJ (2009). Moderate-duration static stretch reduces active and passive plantar flexor moment but not Achilles tendon stiffness or active muscle length. J Appl Physiol.

[22] Koo TK, Li MY (2016). A guideline of selecting and reporting intraclass correlation coefficients for reliability research. J Chiropr Med.

[23] Kruse A, Schranz C, Svehlik M (2017). Mechanical muscle and tendon properties of the plantar flexors are altered even in highly functional children with spastic cerebral palsy. Clin Biomech.

[24] Kruse A, Stafilidis S, Tilp M (2017). Ultrasound and magnetic resonance imaging are not interchangeable to assess the Achilles tendon cross-sectional-area. Eur J Appl Physiol.

[25] Kruse A, Schranz C, Tilp M (2018). Muscle and tendon morphology alterations in children and adolescents with mild forms of spastic cerebral palsy. BMC Pediatr.

[26] Kubo K, Kanehisa H, Fukunaga T (2002). Effects of resistance and stretching training programmes on the viscoelastic properties of human tendon structures in vivo. The J Physiol.

[27] Matsukiyo A, Goh A-C, Asagai Y (2017). Relationship between muscle–tendon length, range of motion, and resistance to passive movement in children with normal and increased tone. J Phys Ther Sci.

[28] Merza E, Pearson S, Lichtwark G (2021). Reliability of human Achilles tendon stiffness measures using freehand 3-D ultrasound. Ultrasound Med Biol.

[29] Nakamura M, Ikezoe T, Takeno Y (2011). Acute and prolonged effect of static stretching on the passive stiffness of the human gastrocnemius muscle tendon unit in vivo. J Orthop Res.

[30] O’Connor PJ, Grainger AJ, Morgan SR (2004). Ultrasound assessment of tendons in asymptomatic volunteers: a study of reproducibility. Eur Radiol.

[31] Ozçakar L, Tok F, de Muynck M (2012). Musculoskeletal ultrasonography in physical and rehabilitation medicine. J Rehabil Med.

[32] Rasmussen OS (2000). Sonography of tendons. Scand J Med Sci Sports.

[33] Ryan ED, Rosenberg JG, Scharville MJ (2013). Test-retest reliability and the minimal detectable change for Achilles tendon length: a panoramic ultrasound assessment. Ultrasound Med Biol.

[34] Shrout PE, Fleiss JL (1979). Intraclass correlations: uses in assessing rater reliability. Psychol Bull.

[35] Silbernagel KG, Shelley K, Powell S (2016). Extended field of view ultrasound imaging to evaluate Achilles tendon length and thickness: a reliability and validity study. Muscles Ligaments Tendons J.

[36] Silbernagel KG, Steele R, Manal K (2012). Deficits in heel-rise height and Achilles tendon elongation occur in patients recovering from an Achilles tendon rupture. Am J Sports Med.

[37] Skypala J, Jandacka D, Hamill J (2019). Reliability of a measurement technique for Achilles tendon length. J Sports Sci.

[38] Stokes OM, Theobald PS, Pugh ND (2010). Panoramic ultrasound to measure in vivo tendo Achilles strain. Foot Ankle Int.

[39] Theis N, Korff T, Kairon H (2013). Does acute passive stretching increase muscle length in children with cerebral palsy?. Clin Biomech.

[40] Valera-Calero JA, Ojedo-Martín C, Fernández-de-Las-Peñas C (2021). Reliability and validity of panoramic ultrasound imaging for evaluating muscular quality and morphology: a systematic review. Ultrasound Med Biol.

[41] Walter SD, Eliasziw M, Donner A (1998). Sample size and optimal designs for reliability studies. Statist Med.

[42] Weide G, Huijing PA, Becher JG (2020). Foot flexibility confounds the assessment of triceps surae extensibility in children with spastic paresis during typical physical examinations. J Biomech.

[43] Weng L, Tirumalai AP, Lowery CM (1997). US extended-field-of-view imaging technology. Radiology.

[44] Ying M, Sin M-H (2005). Comparison of extended field of view and dual image ultrasound techniques: accuracy and reliability of distance measurements in phantom study. Ultrasound Med Biol.

