# Supplementary material for: Validity and reliability of a novel 3D ultrasound approach to assess static lengths and the lengthening behavior of the gastrocnemius medialis muscle and the Achilles tendon in vivo
Source: Knee Surg Sports Traumatol Arthrosc. 2022 Jul 29;30(12):4203–13. doi: 10.1007/s00167-022-07076-2 (PMC9668947; doi:10.1007/s00167-022-07076-2)
Supplement: Supplementary file 1 — (PDF 79 KB) [file 167_2022_7076_MOESM1_ESM.pdf]

Table of contents

\\KFU
Externe Kooperationen
2020
114_MT02_USUS
FastView
lowleg_localizer_1_cor
lowleg_localizer_2_sag
anat-T1w_acq-spacecs7_run-ALL
anat-T1w_acq-spacecs7_run-top
anat-T1w_acq-spacecs7_run-bot

\\KFU\Externe Kooperationen\2020\114_MT02_USUS\FastView

TA: 9 sec Coil Selection: Auto Voxel Size: 5.0×5.0×5.0 mm³ Rel. SNR: 1.00

Properties

Start measurement without further preparation	On
Wait for User to Start	Off
Start measurements	Single Measurement
Prio Recon	Off
Auto Open Inline Display	Off
Auto Close Inline Display	Off
Load Images to MR View&GO	On
Auto Store Images	On
Load Images to Stamp Segments	Off
Load Images to Graphic Segments	Off
Graphic segment	Default
Inline Movie	Off

Routine

Slice Group	1
Slices	1
Distance Factor	100 %
Position	L0.0 A33.0 F132.2 mm
Orientation	Transversal
Phase Encoding Dir.	A >> P
FoV Read	480 mm
FoV Phase	87.5 %
Slice Thickness	5.0 mm
TR	2.6 ms
TE	1.44 ms
AutoAlign	---
Coil Elements	BC

Contrast - Common

TR	2.6 ms
TE	1.44 ms
Fat-Water Contrast	Standard

Contrast - Dynamic

Dynamic Mode	Standard

Resolution - Common

FoV Read	480 mm
FoV Phase	87.5 %
Slice Thickness	5.0 mm
Base Resolution	96
Phase Resolution	100 %

Resolution - Acceleration

Phase Partial Fourier	6/8

Resolution - Filter

Normalize	Off

Geometry - Common

Slice Group	1
Slices	1
Distance Factor	100 %
Position	L0.0 A33.0 F132.2 mm
Orientation	Transversal
Phase Encoding Dir.	A >> P
FoV Read	480 mm
FoV Phase	87.5 %
Slice Thickness	5.0 mm

Geometry - Common

TR	2.6 ms

Geometry - AutoAlign

Slice Group	1
Position	L0.0 A33.0 F132.2 mm
Orientation	Transversal
Phase Encoding Dir.	A >> P
AutoAlign	---
Initial Position	L0.0 A33.0 F151.2
L	0.0 mm
A	33.0 mm
F	151.2 mm
Initial Orientation	Transversal
Initial Rotation	0.00 deg

Geometry - FastView

Range Start	132 mm
Range Start	F
Total FoV	424 mm
Total FoV	F >> H
Slices	1
Slice Thickness	5.0 mm
Distance Factor	100 %
FoV Read	480 mm
FoV Phase	87.5 %
Table Speed	46.00 mm/s
FastView Adjustments	On
B0 Shim	Tune up

Geometry - Tim Planning Suite

Table Position	132 mm
Table Position	F
Inline Composing	Off

System - Miscellaneous

Coil Selection	Auto Coil Select
MSMA	S - C - T
Sagittal	R >> L
Coronal	A >> P
Transversal	F >> H
Matrix Optimization	Off

System - Adjustments

Adjustment Strategy	Standard
B0 Shim	Tune up
CoilShim	Off
Adjustment Tolerance	Maximum
Adjust with Body Coil	On
Confirm Frequency	Never
Assume Silicone	Off
Adj. Water Suppr.	Off
FastView Adjustments	On

System - Tx/Rx

Frequency 1H	123.262898 MHz
Correction Factor	1.00
Image Scaling	1.000

Sequence - Part 1

Sequence Name	flct

Sequence - Part 1

Dimension	2D
Bandwidth	801 Hz/Px

Sequence - Assistant

SAR Assistant	Off

\\KFU\Externe Kooperationen\2020\114_MT02_USUS\lowleg_localizer_1_cor

TA: 37 sec Coil Selection: Auto Voxel Size: 1.7×1.7×6.0 mm³ Acc:: 3 Rel. SNR: 1.00

Properties

Start measurement without further preparation	Off
Wait for User to Start	On
Start measurements	Single Measurement
Prio Recon	Off
Auto Open Inline Display	Off
Auto Close Inline Display	Off
Load Images to MR View&GO	On
Auto Store Images	On
Load Images to Stamp Segments	On
Load Images to Graphic Segments	On
Graphic segment	Default
Inline Movie	Off

Routine

Slice Group	1
Slices	25
Distance Factor	100 %
Position	L6.6 P50.6 H350.1 mm
Orientation	Coronal
Phase Encoding Dir.	F >> H
Phase Oversampling	70 %
FoV Read	550 mm
FoV Phase	100.0 %
Slice Thickness	6.0 mm
TR	1400.0 ms
TE	88.00 ms
Averages	1
Concatenations	3
AutoAlign	---
Coil Elements	BO1-3;HE4;NE2;SP1-3

Contrast - Common

TR	1400.0 ms
TE	88.00 ms
TD	0.00 ms
MTC	Off
Magn. Preparation	None
Flip Angle	160 deg
Fat-Water Contrast	Standard
Dark Blood	Off
Contrasts	1
Wrap-up Magn.	None
Reconstruction	Magnitude

Contrast - Dynamic

Dynamic Mode	Standard
Measurements	1
Multiple Series	Off

Resolution - Common

FoV Read	550 mm
FoV Phase	100.0 %
Slice Thickness	6.0 mm
Base Resolution	320
Phase Resolution	80 %
Interpolation	Off

Resolution - Acceleration

Acceleration mode	GRAPPA

Resolution - Acceleration

Reference Scans	TSE/Separate
Acceleration Factor PE	3
Reference Lines PE	36
Phase Partial Fourier	5/8

Resolution - Filter

Raw Filter	Off
Elliptical Filter	Off
Distortion Correction	2D
Normalize	Prescan
Image Filter	Off

Geometry - Common

Slice Group	1
Slices	25
Distance Factor	100 %
Position	L6.6 P50.6 H350.1 mm
Orientation	Coronal
Phase Encoding Dir.	F >> H
Phase Oversampling	70 %
FoV Read	550 mm
FoV Phase	100.0 %
Slice Thickness	6.0 mm
TR	1400.0 ms
Multi-Slice Mode	Single Shot
Series	Interleaved
Concatenations	3

Geometry - AutoAlign

Slice Group	1
Position	L6.6 P50.6 H350.1 mm
Orientation	Coronal
Phase Encoding Dir.	F >> H
AutoAlign	---
Initial Position	L6.6 P50.6 H350.1
L	6.6 mm
P	50.6 mm
H	350.1 mm
Initial Orientation	Coronal
Initial Rotation	90.00 deg

Geometry - Navigator

Geometry - Saturation

Special Saturation	None

Geometry - Tim Planning Suite

Set-n-Go Protocol	Off
Table Position	350 mm
Table Position	H
Inline Composing	Off

System - Miscellaneous

Coil Selection	Auto Coil Select
MSMA	S - C - T
Sagittal	R >> L
Coronal	A >> P
Transversal	H >> F
Coil Combination	Adaptive Combine
Matrix Optimization	Off

System - Miscellaneous

Coil Focus	Flat

System - Adjustments

Adjustment Strategy	Standard
B0 Shim	Tune up
B1 Shim	TrueForm
CoilShim	Off
Adjustment Tolerance	Auto
Adjust with Body Coil	Off
Confirm Frequency	Never
Assume Silicone	Off

System - Adjust Volume

Position	Isocenter
Orientation	Transversal
Rotation	0.00 deg
A >> P	263 mm
R >> L	350 mm
F >> H	350 mm
Reset	Off

System - Tx/Rx

Frequency 1H	123.262898 MHz
? Ref. Amplitude 1H	0.000 V
Reset	Off
Correction Factor	1.00
Image Scaling	1.000

Physio - Signal

1st Signal/Mode	None
TR	1400.0 ms
Concatenations	3

Physio - Cardiac

Fat-Water Contrast	Standard
Magn. Preparation	None
Dark Blood	Off
FoV Read	550 mm
FoV Phase	100.0 %
Phase Resolution	80 %
Dynamic Mode	Standard

Physio - PACE

Resp. Control	Off
Concatenations	3

Inline - Subtraction

Subtract	Off
Measurements	1
StdDev	Off
Save Original Images	On

Inline - Cardiac

Magn. Preparation	None
Save Original Images	On
Contrasts	1
TE	88.00 ms
TR	1400.0 ms

Inline - MIP

MIP Sag	Off
MIP Cor	Off
MIP Tra	Off

Inline - MIP

MIP Time	Off
Radial MIP	Off
Save Original Images	On
MPR Sag	Off
MPR Cor	Off
MPR Tra	Off

Inline - Composing

Inline Composing	Off

Sequence - Part 1

Sequence Name	h
Dimension	2D
RF Pulse Type	Normal
Gradient Mode	Fast
Flow Compensation	None
Bandwidth	679 Hz/Px
Echo Spacing	5.20 ms
Turbo Factor	256

Sequence - Part 2

Introduction	On
Hyperecho	Off

Sequence - Assistant

SAR Assistant	Flip Angle
Min Flip Angle	120 deg
Allowed Delay	30 s

\\KFU\Externe Kooperationen\2020\114_MT02_USUS\lowleg_localizer_2_sag
TA: 58 sec Coil Selection: Auto Voxel Size: 1.7×1.7×6.0 mm³ Acc:: 3 Rel. SNR: 1.00

Properties

Start measurement without further preparation	Off
Wait for User to Start	On
Start measurements	Single Measurement
Prio Recon	Off
Auto Open Inline Display	Off
Auto Close Inline Display	Off
Load Images to MR View&GO	On
Auto Store Images	On
Load Images to Stamp Segments	On
Load Images to Graphic Segments	On
Graphic segment	Default
Inline Movie	Off

Routine

Slice Group	1
Slices	40
Distance Factor	60 %
Position	R2.1 A19.2 H319.9 mm
Orientation	Sagittal
Phase Encoding Dir.	A >> P
Phase Oversampling	70 %
FoV Read	550 mm
FoV Phase	75.0 %
Slice Thickness	6.0 mm
TR	1400.0 ms
TE	88.00 ms
Averages	1
Concatenations	3
AutoAlign	---
Coil Elements	BO1-3;HE4;NE2;SP1-3

Contrast - Common

TR	1400.0 ms
TE	88.00 ms
TD	0.00 ms
MTC	Off
Magn. Preparation	None
Flip Angle	160 deg
Fat-Water Contrast	Standard
Dark Blood	Off
Contrasts	1
Wrap-up Magn.	None
Reconstruction	Magnitude

Contrast - Dynamic

Dynamic Mode	Standard
Measurements	1
Multiple Series	Off

Resolution - Common

FoV Read	550 mm
FoV Phase	75.0 %
Slice Thickness	6.0 mm
Base Resolution	320
Phase Resolution	80 %
Interpolation	Off

Resolution - Acceleration

Acceleration mode	GRAPPA

Resolution - Acceleration

Reference Scans	TSE/Separate
Acceleration Factor PE	3
Reference Lines PE	36
Phase Partial Fourier	5/8

Resolution - Filter

Raw Filter	Off
Elliptical Filter	Off
Distortion Correction	2D
Normalize	Prescan
Image Filter	Off

Geometry - Common

Slice Group	1
Slices	40
Distance Factor	60 %
Position	R2.1 A19.2 H319.9 mm
Orientation	Sagittal
Phase Encoding Dir.	A >> P
Phase Oversampling	70 %
FoV Read	550 mm
FoV Phase	75.0 %
Slice Thickness	6.0 mm
TR	1400.0 ms
Multi-Slice Mode	Single Shot
Series	Interleaved
Concatenations	3

Geometry - AutoAlign

Slice Group	1
Position	R2.1 A19.2 H319.9 mm
Orientation	Sagittal
Phase Encoding Dir.	A >> P
AutoAlign	---
Initial Position	R2.1 A19.2 H319.9
R	2.1 mm
A	19.2 mm
H	319.9 mm
Initial Orientation	Sagittal
Initial Rotation	0.00 deg

Geometry - Navigator

Geometry - Saturation

Special Saturation	None

Geometry - Tim Planning Suite

Set-n-Go Protocol	Off
Table Position	320 mm
Table Position	H
Inline Composing	Off

System - Miscellaneous

Coil Selection	Auto Coil Select
MSMA	S - C - T
Sagittal	R >> L
Coronal	A >> P
Transversal	H >> F
Coil Combination	Adaptive Combine
Matrix Optimization	Off

System - Miscellaneous

Coil Focus	Flat

System - Adjustments

Adjustment Strategy	Standard
B0 Shim	Tune up
B1 Shim	TrueForm
CoilShim	Off
Adjustment Tolerance	Auto
Adjust with Body Coil	Off
Confirm Frequency	Never
Assume Silicone	Off

System - Adjust Volume

Position	Isocenter
Orientation	Transversal
Rotation	0.00 deg
A >> P	263 mm
R >> L	350 mm
F >> H	350 mm
Reset	Off

System - Tx/Rx

Frequency 1H	123.262898 MHz
? Ref. Amplitude 1H	0.000 V
Reset	Off
Correction Factor	1.00
Image Scaling	1.000

Physio - Signal

1st Signal/Mode	None
TR	1400.0 ms
Concatenations	3

Physio - Cardiac

Fat-Water Contrast	Standard
Magn. Preparation	None
Dark Blood	Off
FoV Read	550 mm
FoV Phase	75.0 %
Phase Resolution	80 %
Dynamic Mode	Standard

Physio - PACE

Resp. Control	Off
Concatenations	3

Inline - Subtraction

Subtract	Off
Measurements	1
StdDev	Off
Save Original Images	On

Inline - Cardiac

Magn. Preparation	None
Save Original Images	On
Contrasts	1
TE	88.00 ms
TR	1400.0 ms

Inline - MIP

MIP Sag	Off
MIP Cor	Off
MIP Tra	Off

Inline - MIP

MIP Time	Off
Radial MIP	Off
Save Original Images	On
MPR Sag	Off
MPR Cor	Off
MPR Tra	Off

Inline - Composing

Inline Composing	Off

Sequence - Part 1

Sequence Name	h
Dimension	2D
RF Pulse Type	Normal
Gradient Mode	Fast
Flow Compensation	None
Bandwidth	679 Hz/Px
Echo Spacing	5.20 ms
Turbo Factor	192

Sequence - Part 2

Introduction	On
Hyperecho	Off

Sequence - Assistant

SAR Assistant	Flip Angle
Min Flip Angle	120 deg
Allowed Delay	30 s

\\KFU\Externe Kooperationen\2020\114_MT02_USUS\anat-T1w_acq-spacecs7_run-ALL

TA: 5:28 min Coil Selection: Auto Voxel Size: 0.5×0.5×1.0 mm³ Acc:: 7.0 Rel. SNR: 1.00

Properties

Start measurement without further preparation	On
Wait for User to Start	On
Start measurements	Single Measurement
Prio Recon	Off
Auto Open Inline Display	Off
Auto Close Inline Display	Off
Load Images to MR View&GO	On
Auto Store Images	On
Load Images to Stamp Segments	On
Load Images to Graphic Segments	On
Graphic segment	Default
Inline Movie	Off

Routine

Slab Group	1
Slabs	1
Position	L0.8 P9.9 H279.0 mm
Orientation	Sagittal
Phase Encoding Dir.	A >> P
Slices per Slab	288
Phase Oversampling	0 %
Slice Oversampling	0.0 %
FoV Read	544 mm
FoV Phase	80.1 %
Slice Thickness	1.00 mm
TR	638.0 ms
TE	16.00 ms
Averages	1.0
Concatenations	1
AutoAlign	Knee > Standard
Coil Elements	BO1-3;HE2,4;NE2;SP1-3

Contrast - Common

TR	638.0 ms
TE	16.00 ms
MTC	Off
Magn. Preparation	None
Flip Angle Mode	T1 Var
Fat-Water Contrast	Standard
Dark Blood	Off
Blood Suppression	Off
Wrap-up Magn.	Restore
Reconstruction	Magnitude

Contrast - Dynamic

Dynamic Mode	Standard
Measurements	1
Multiple Series	Each Measurement
Reordering	Radial

Resolution - Common

FoV Read	544 mm
FoV Phase	80.1 %
Slice Thickness	1.00 mm
Base Resolution	544
Phase Resolution	100 %
Slice Resolution	100 %
Interpolation	On

Resolution - Acceleration

Acceleration mode	CS
Total Factor	7.0
Reference Scans	Integrated
Reference Lines PE	24
Reference Lines 3D	24
Phase Partial Fourier	Off
Slice Partial Fourier	Off
Elliptical Scanning	Off

Resolution - Filter

Raw Filter	Off
Elliptical Filter	On
Distortion Correction	2D
Normalize	Prescan
Image Filter	Off

Geometry - Common

Slab Group	1
Slabs	1
Position	L0.8 P9.9 H279.0 mm
Orientation	Sagittal
Phase Encoding Dir.	A >> P
Slices per Slab	288
Phase Oversampling	0 %
Slice Oversampling	0.0 %
FoV Read	544 mm
FoV Phase	80.1 %
Slice Thickness	1.00 mm
TR	638.0 ms
Concatenations	1

Geometry - AutoAlign

Slab Group	1
Position	L0.8 P9.9 H279.0 mm
Orientation	Sagittal
Phase Encoding Dir.	A >> P
AutoAlign	Knee > Standard
Initial Position	L0.8 P9.9 H279.0
L	0.8 mm
P	9.9 mm
H	279.0 mm
Initial Orientation	Sagittal
Initial Rotation	0.00 deg

Geometry - Navigator

Geometry - Saturation

Special Saturation	None

Geometry - Tim Planning Suite

Set-n-Go Protocol	Off
Table Position	279 mm
Table Position	H
Inline Composing	Off

System - Miscellaneous

Coil Selection	Auto Coil Select
MSMA	S - C - T
Sagittal	R >> L
Coronal	A >> P

System - Miscellaneous

Transversal	H >> F
Coil Combination	Adaptive Combine
Matrix Optimization	Performance
Coil Focus	Flat

System - Adjustments

Adjustment Strategy	Standard
B0 Shim	Standard
B1 Shim	TrueForm
CoilShim	Off
Adjustment Tolerance	Auto
Adjust with Body Coil	Off
Confirm Frequency	Never
Assume Silicone	Off

System - Adjust Volume

Position	L0.8 P9.9 H279.0 mm
Orientation	Sagittal
Rotation	0.00 deg
A >> P	436 mm
F >> H	544 mm
R >> L	288 mm
Reset	Off

System - Tx/Rx

Frequency 1H	123.262898 MHz
? Ref. Amplitude 1H	0.000 V
Reset	Off
Correction Factor	1.00
Image Scaling	1.000

Physio - Signal

1st Signal/Mode	None
Trigger Delay	0 ms
TR	638.0 ms
Concatenations	1

Physio - Cardiac

Fat-Water Contrast	Standard
Magn. Preparation	None
Dark Blood	Off
FoV Read	544 mm
FoV Phase	80.1 %
Phase Resolution	100 %
Dynamic Mode	Standard

Physio - PACE

Resp. Control	Off
Concatenations	1

Inline - Subtraction

Subtract	Off
Measurements	1
StdDev	Off
Save Original Images	On

Inline - Cardiac

Magn. Preparation	None
Save Original Images	On
TE	16.00 ms
TR	638.0 ms

Inline - MIP

MIP Sag	Off
MIP Cor	Off
MIP Tra	Off
MIP Time	Off
Radial MIP	Off
Save Original Images	On
MPR Sag	Off
MPR Cor	Off
MPR Tra	Off

Inline - Composing

Inline Composing	Off

Sequence - Part 1

Sequence Name	spcR
Dimension	3D
Excitation	Non-sel.
RF Pulse Type	Fast
Gradient Mode	Fast
Flow Compensation	None
Reordering	Radial
Bandwidth	511 Hz/Px
Echo Spacing	4.08 ms
Turbo Factor	40
Echo Train Duration	167 ms

Sequence - Part 2

Introduction	On

Sequence - Assistant

SAR Assistant	Off
Allowed Delay	30 s

\\KFU\Externe Kooperationen\2020\114_MT02_USUS\anat-T1w_acq-spacecs7_run-top
TA: 5:28 min Coil Selection: Auto Voxel Size: 0.5×0.5×1.0 mm³ Acc:: 7.0 Rel. SNR: 1.00

Properties

Start measurement without further preparation	On
Wait for User to Start	On
Start measurements	Single Measurement
Prio Recon	Off
Auto Open Inline Display	Off
Auto Close Inline Display	Off
Load Images to MR View&GO	On
Auto Store Images	On
Load Images to Stamp Segments	On
Load Images to Graphic Segments	On
Graphic segment	Default
Inline Movie	Off

Routine

Slab Group	1
Slabs	1
Position	L0.8 P9.9 H279.0 mm
Orientation	Sagittal
Phase Encoding Dir.	A >> P
Slices per Slab	288
Phase Oversampling	0 %
Slice Oversampling	0.0 %
FoV Read	544 mm
FoV Phase	80.1 %
Slice Thickness	1.00 mm
TR	638.0 ms
TE	16.00 ms
Averages	1.0
Concatenations	1
AutoAlign	Knee > Standard
Coil Elements	BO1-3;HE2,4;NE2;SP1-3

Contrast - Common

TR	638.0 ms
TE	16.00 ms
MTC	Off
Magn. Preparation	None
Flip Angle Mode	T1 Var
Fat-Water Contrast	Standard
Dark Blood	Off
Blood Suppression	Off
Wrap-up Magn.	Restore
Reconstruction	Magnitude

Contrast - Dynamic

Dynamic Mode	Standard
Measurements	1
Multiple Series	Each Measurement
Reordering	Radial

Resolution - Common

FoV Read	544 mm
FoV Phase	80.1 %
Slice Thickness	1.00 mm
Base Resolution	544
Phase Resolution	100 %
Slice Resolution	100 %
Interpolation	On

Resolution - Acceleration

Acceleration mode	CS
Total Factor	7.0
Reference Scans	Integrated
Reference Lines PE	24
Reference Lines 3D	24
Phase Partial Fourier	Off
Slice Partial Fourier	Off
Elliptical Scanning	Off

Resolution - Filter

Raw Filter	Off
Elliptical Filter	On
Distortion Correction	2D
Normalize	Prescan
Image Filter	Off

Geometry - Common

Slab Group	1
Slabs	1
Position	L0.8 P9.9 H279.0 mm
Orientation	Sagittal
Phase Encoding Dir.	A >> P
Slices per Slab	288
Phase Oversampling	0 %
Slice Oversampling	0.0 %
FoV Read	544 mm
FoV Phase	80.1 %
Slice Thickness	1.00 mm
TR	638.0 ms
Concatenations	1

Geometry - AutoAlign

Slab Group	1
Position	L0.8 P9.9 H279.0 mm
Orientation	Sagittal
Phase Encoding Dir.	A >> P
AutoAlign	Knee > Standard
Initial Position	L0.8 P9.9 H279.0
L	0.8 mm
P	9.9 mm
H	279.0 mm
Initial Orientation	Sagittal
Initial Rotation	0.00 deg

Geometry - Navigator

Geometry - Saturation

Special Saturation	None

Geometry - Tim Planning Suite

Set-n-Go Protocol	Off
Table Position	279 mm
Table Position	H
Inline Composing	Off

System - Miscellaneous

Coil Selection	Auto Coil Select
MSMA	S - C - T
Sagittal	R >> L
Coronal	A >> P

System - Miscellaneous

Transversal	H >> F
Coil Combination	Adaptive Combine
Matrix Optimization	Performance
Coil Focus	Flat

System - Adjustments

Adjustment Strategy	Standard
B0 Shim	Standard
B1 Shim	TrueForm
CoilShim	Off
Adjustment Tolerance	Auto
Adjust with Body Coil	Off
Confirm Frequency	Never
Assume Silicone	Off

System - Adjust Volume

Position	L0.8 P9.9 H279.0 mm
Orientation	Sagittal
Rotation	0.00 deg
A >> P	436 mm
F >> H	544 mm
R >> L	288 mm
Reset	Off

System - Tx/Rx

Frequency 1H	123.262898 MHz
? Ref. Amplitude 1H	0.000 V
Reset	Off
Correction Factor	1.00
Image Scaling	1.000

Physio - Signal

1st Signal/Mode	None
Trigger Delay	0 ms
TR	638.0 ms
Concatenations	1

Physio - Cardiac

Fat-Water Contrast	Standard
Magn. Preparation	None
Dark Blood	Off
FoV Read	544 mm
FoV Phase	80.1 %
Phase Resolution	100 %
Dynamic Mode	Standard

Physio - PACE

Resp. Control	Off
Concatenations	1

Inline - Subtraction

Subtract	Off
Measurements	1
StdDev	Off
Save Original Images	On

Inline - Cardiac

Magn. Preparation	None
Save Original Images	On
TE	16.00 ms
TR	638.0 ms

Inline - MIP

MIP Sag	Off
MIP Cor	Off
MIP Tra	Off
MIP Time	Off
Radial MIP	Off
Save Original Images	On
MPR Sag	Off
MPR Cor	Off
MPR Tra	Off

Inline - Composing

Inline Composing	Off

Sequence - Part 1

Sequence Name	spcR
Dimension	3D
Excitation	Non-sel.
RF Pulse Type	Fast
Gradient Mode	Fast
Flow Compensation	None
Reordering	Radial
Bandwidth	511 Hz/Px
Echo Spacing	4.08 ms
Turbo Factor	40
Echo Train Duration	167 ms

Sequence - Part 2

Introduction	On

Sequence - Assistant

SAR Assistant	Off
Allowed Delay	30 s

\\KFU\Externe Kooperationen\2020\114_MT02_USUS\anat-T1w_acq-spacecs7_run-bot
TA: 5:28 min Coil Selection: Auto Voxel Size: 0.5×0.5×1.0 mm³ Acc:: 7.0 Rel. SNR: 1.00

Properties

Start measurement without further preparation	On
Wait for User to Start	On
Start measurements	Single Measurement
Prio Recon	Off
Auto Open Inline Display	Off
Auto Close Inline Display	Off
Load Images to MR View&GO	On
Auto Store Images	On
Load Images to Stamp Segments	On
Load Images to Graphic Segments	On
Graphic segment	Default
Inline Movie	Off

Routine

Slab Group	1
Slabs	1
Position	L0.8 P9.9 H279.0 mm
Orientation	Sagittal
Phase Encoding Dir.	A >> P
Slices per Slab	288
Phase Oversampling	0 %
Slice Oversampling	0.0 %
FoV Read	544 mm
FoV Phase	80.1 %
Slice Thickness	1.00 mm
TR	638.0 ms
TE	16.00 ms
Averages	1.0
Concatenations	1
AutoAlign	Knee > Standard
Coil Elements	BO1-3;HE2,4;NE2;SP1-3

Contrast - Common

TR	638.0 ms
TE	16.00 ms
MTC	Off
Magn. Preparation	None
Flip Angle Mode	T1 Var
Fat-Water Contrast	Standard
Dark Blood	Off
Blood Suppression	Off
Wrap-up Magn.	Restore
Reconstruction	Magnitude

Contrast - Dynamic

Dynamic Mode	Standard
Measurements	1
Multiple Series	Each Measurement
Reordering	Radial

Resolution - Common

FoV Read	544 mm
FoV Phase	80.1 %
Slice Thickness	1.00 mm
Base Resolution	544
Phase Resolution	100 %
Slice Resolution	100 %
Interpolation	On

Resolution - Acceleration

Acceleration mode	CS
Total Factor	7.0
Reference Scans	Integrated
Reference Lines PE	24
Reference Lines 3D	24
Phase Partial Fourier	Off
Slice Partial Fourier	Off
Elliptical Scanning	Off

Resolution - Filter

Raw Filter	Off
Elliptical Filter	On
Distortion Correction	2D
Normalize	Prescan
Image Filter	Off

Geometry - Common

Slab Group	1
Slabs	1
Position	L0.8 P9.9 H279.0 mm
Orientation	Sagittal
Phase Encoding Dir.	A >> P
Slices per Slab	288
Phase Oversampling	0 %
Slice Oversampling	0.0 %
FoV Read	544 mm
FoV Phase	80.1 %
Slice Thickness	1.00 mm
TR	638.0 ms
Concatenations	1

Geometry - AutoAlign

Slab Group	1
Position	L0.8 P9.9 H279.0 mm
Orientation	Sagittal
Phase Encoding Dir.	A >> P
AutoAlign	Knee > Standard
Initial Position	L0.8 P9.9 H279.0
L	0.8 mm
P	9.9 mm
H	279.0 mm
Initial Orientation	Sagittal
Initial Rotation	0.00 deg

Geometry - Navigator

Geometry - Saturation

Special Saturation	None

Geometry - Tim Planning Suite

Set-n-Go Protocol	Off
Table Position	279 mm
Table Position	H
Inline Composing	Off

System - Miscellaneous

Coil Selection	Auto Coil Select
MSMA	S - C - T
Sagittal	R >> L
Coronal	A >> P

System - Miscellaneous

Transversal	H >> F
Coil Combination	Adaptive Combine
Matrix Optimization	Performance
Coil Focus	Flat

System - Adjustments

Adjustment Strategy	Standard
B0 Shim	Standard
B1 Shim	TrueForm
CoilShim	Off
Adjustment Tolerance	Auto
Adjust with Body Coil	Off
Confirm Frequency	Never
Assume Silicone	Off

System - Adjust Volume

Position	L0.8 P9.9 H279.0 mm
Orientation	Sagittal
Rotation	0.00 deg
A >> P	436 mm
F >> H	544 mm
R >> L	288 mm
Reset	Off

System - Tx/Rx

Frequency 1H	123.262898 MHz
? Ref. Amplitude 1H	0.000 V
Reset	Off
Correction Factor	1.00
Image Scaling	1.000

Physio - Signal

1st Signal/Mode	None
Trigger Delay	0 ms
TR	638.0 ms
Concatenations	1

Physio - Cardiac

Fat-Water Contrast	Standard
Magn. Preparation	None
Dark Blood	Off
FoV Read	544 mm
FoV Phase	80.1 %
Phase Resolution	100 %
Dynamic Mode	Standard

Physio - PACE

Resp. Control	Off
Concatenations	1

Inline - Subtraction

Subtract	Off
Measurements	1
StdDev	Off
Save Original Images	On

Inline - Cardiac

Magn. Preparation	None
Save Original Images	On
TE	16.00 ms
TR	638.0 ms

Inline - MIP

MIP Sag	Off
MIP Cor	Off
MIP Tra	Off
MIP Time	Off
Radial MIP	Off
Save Original Images	On
MPR Sag	Off
MPR Cor	Off
MPR Tra	Off

Inline - Composing

Inline Composing	Off

Sequence - Part 1

Sequence Name	spcR
Dimension	3D
Excitation	Non-sel.
RF Pulse Type	Fast
Gradient Mode	Fast
Flow Compensation	None
Reordering	Radial
Bandwidth	511 Hz/Px
Echo Spacing	4.08 ms
Turbo Factor	40
Echo Train Duration	167 ms

Sequence - Part 2

Introduction	On

Sequence - Assistant

SAR Assistant	Off
Allowed Delay	30 s
