# Supplementary figures and images for: Validity and reliability of a novel 3D ultrasound approach to assess static lengths and the lengthening behavior of the gastrocnemius medialis muscle and the Achilles tendon in vivo
Source: Knee Surg Sports Traumatol Arthrosc. 2022 Jul 29;30(12):4203–13. doi: 10.1007/s00167-022-07076-2 (PMC9668947; doi:10.1007/s00167-022-07076-2)

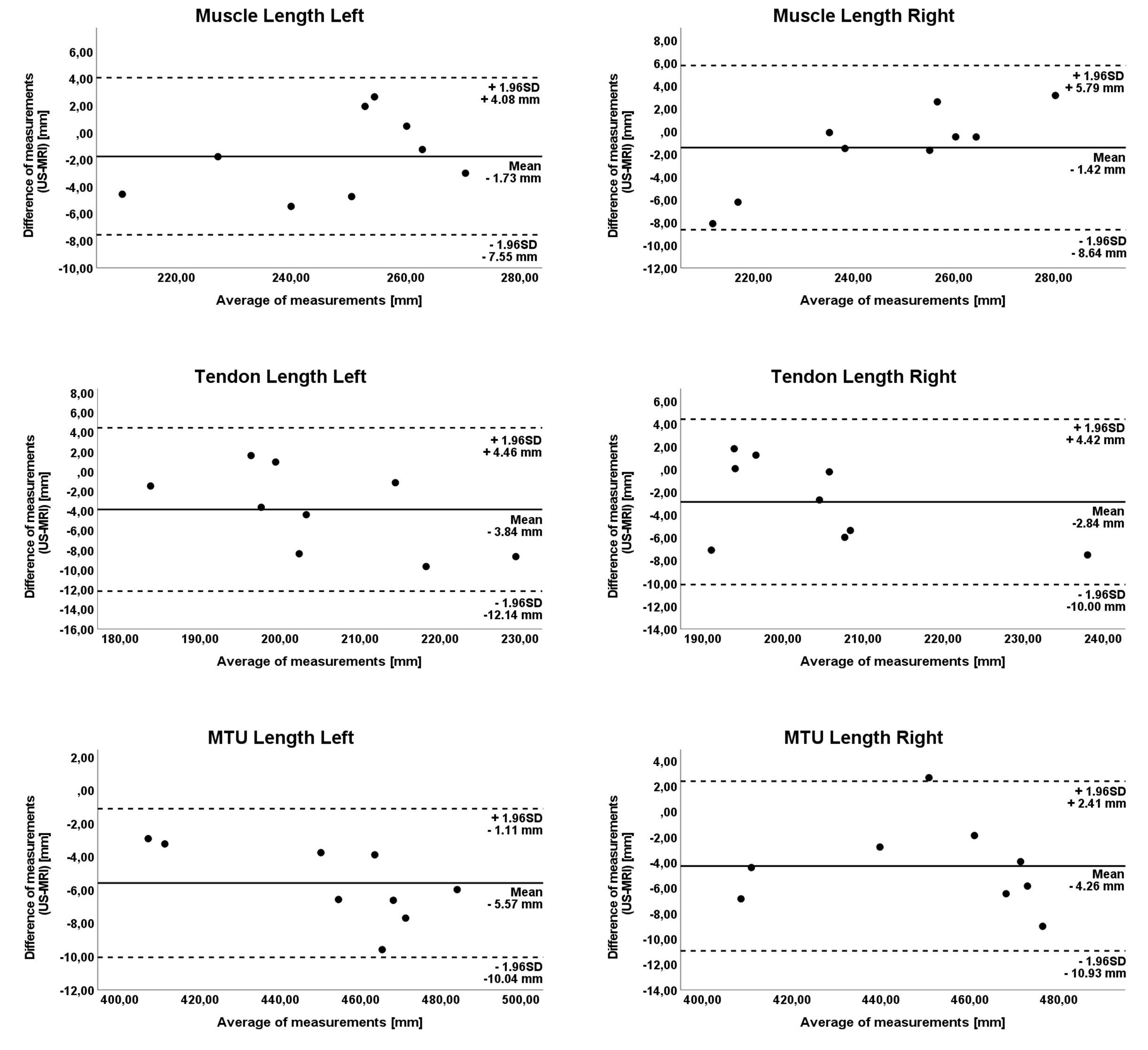

Supplement: Supplementary file 2 — (PNG 564 KB) [file 167_2022_7076_MOESM2_ESM.png]
