# Supplementary material for: Validity and reliability of a novel 3D ultrasound approach to assess static lengths and the lengthening behavior of the gastrocnemius medialis muscle and the Achilles tendon in vivo
Source: Knee Surg Sports Traumatol Arthrosc. 2022 Jul 29;30(12):4203–13. doi: 10.1007/s00167-022-07076-2 (PMC9668947; doi:10.1007/s00167-022-07076-2)
Supplement: Supplementary file 3 — (DOCX 13 KB) [file 167_2022_7076_MOESM3_ESM.docx]

**Appendix**

**Figure.** Difference between ultrasound (US) and magnetic resonance imaging (MRI) assessments of the gastrocnemius medialis muscle-tendon unit (MTU) length, gastrocnemius medialis muscle belly length, and Achilles tendon length. Bland-Altman plots showing the difference of the ultrasound and MRI measurements against their mean. SD = standard deviation.
